# Implementation and evaluation of a shock curriculum using simulation in Manila, Philippines: a prospective cohort study

**DOI:** 10.1186/s12909-022-03669-0

**Published:** 2022-08-05

**Authors:** Sarah E. Gardner Yelton, Lorelie Cañete Ramos, Carolyn J. Reuland, Paula Pilar G. Evangelista, Nicole A. Shilkofski

**Affiliations:** 1 Department of Anesthesiology and Critical Care Medicine, Charlotte R. Bloomberg Children’s Center, Johns Hopkins University School of Medicine, 1800 Orleans Street, Room 6349 D1, Baltimore, MD 21287 USA; 2 Department of Pediatric Critical Care, Philippine Children’s Medical Center, Quezon City, Philippines; 3 Johns Hopkins University School of Medicine, Baltimore, MD USA; 4 Department of Pediatrics, Charlotte R. Bloomberg Children’s Center, Johns Hopkins University School of Medicine, Baltimore, MD USA

**Keywords:** Global health, Simulation, Shock, Children, Medical education

## Abstract

**Background:**

Shock causes significant morbidity and mortality in children living in resource-limited settings. Simulation has been successfully used as an educational tool for medical professionals internationally. We sought to improve comfort and knowledge regarding shock recognition and fluid management by implementing a pediatric shock curriculum using simulation as an assessment for trainees in Manila, Philippines.

**Methods:**

We assessed a shock curriculum focused on patients with malnutrition in a prospective cohort study, using a written test and a videotaped simulation-based objective standardized clinical examination. Implementation occurred in March 2020 with 24 Filipino pediatric residents at a single institution in Manila. Outcomes included time to initiation of fluid resuscitation, improvement in confidence, knowledge on a written assessment, and performance in simulation. Results were compared pre- and post-intervention using Wilcoxon signed-rank test.

**Results:**

The time to initiation of fluids did not change between the baseline simulation (median [interquartile range] = 71.5 seconds [52–116.5]) and the final simulation (68 seconds [52.5–89]; *P* = 0.42). Confidence in identifying shock and malnutrition, managing hypovolemic shock, managing septic shock, and placing intraosseous access all increased (*P* < 0.01) post-intervention. Written test scores showed no improvement, but performance in simulation, measured using a checklist, improved from a total score of 10 [8.5–11] to 15 [13-16] (*P* < 0.01).

**Conclusion:**

In our study of a simulation-based shock education program, we showed improvement in confidence and knowledge as measured by a resuscitation checklist. It is feasible to establish a successful simulation-based education program in a low-resource setting.

**Supplementary Information:**

The online version contains supplementary material available at 10.1186/s12909-022-03669-0.

## Background

Children living in resource-limited settings (RLS) suffer disproportionate morbidity and mortality secondary to common childhood illnesses. In 2019, over 1 million children worldwide died from sepsis, diarrhea or pneumonia [1]. Despite barriers including limited training in pediatric-specific care and lack of funding, healthcare providers in RLS must treat children in shock. Simulation has been shown to effectively educate trainees on fluid resuscitation in shock. In studies of simulation-based shock education programs, participants have consistently shown improvements with respect to confidence [2] and knowledge [3, 4]. Knowledge acquisition may be measured via written assessment or by performance on a resuscitation checklist, several of which have been validated for various topics from intraosseous (IO) needle placement [5] to shock [6] and cardiopulmonary resuscitation [3, 7–10]. Greater confidence in skills and improvement in knowledge scores can translate into changes in clinical practice although the effect on outcomes is less well-known. In a study by Qian et al., providers who participated in a simulation program on first-hour care of sepsis were more likely to initiate fluids promptly in the clinical setting [11]. Rapid recognition, in addition to initiation of appropriate fluid resuscitation, has been documented to decrease the morbidity and mortality associated with most forms of shock [12, 13], as every hour of delay is associated with 50% increased odds of mortality [14]. Improvement in outcomes may be more likely with frequent re-education sessions to prevent skill decay [3, 6, 15]. Therefore, the World Health Organization (WHO) suggests a low-dose/high-frequency training paradigm for healthcare workers [16].

Teaching fluid resuscitation in RLS is not straightforward. In 2011, the Fluid Expansion as Supportive Therapy (FEAST) trial, which was conducted in three east African nations, was stopped prematurely because children with severe febrile illness who received rapid fluid resuscitation with either 0.9% sodium chloride or 5% albumin boluses had a higher mortality rate than those who received routine care [17]. Subsequently, the WHO and Surviving Sepsis Campaign updated their guidelines to recommend cautious fluid resuscitation in RLS, particularly when critical care is unavailable [16, 18]. One hypothesis for the increased mortality of patients who receive rapid fluid resuscitation in RLS is the higher rate of co-morbid malnutrition. Patients with severe acute malnutrition may have associated structural changes to the myocardium [19, 20] and are at high risk for sudden death, thought to be secondary to associated myocardial dysfunction [21–24]. Thereby, the WHO recommends reserving intravenous fluids in patients with malnutrition for those in decompensated shock [16]. While there have been a handful of studies evaluating the safety of various fluids given to patients with severe acute malnutrition and hypovolemic or septic shock, the data has not been conclusive, emphasizing the importance of repeated patient reevaluation to interventions [25–30]. Nevertheless, fluid administration is still essential. In fact, children in RLS with septic shock have increased mortality if they do not receive fluids within the first 30 minutes of presentation [31]. Hence, in settings with high rates of malnutrition, such as the Philippines, practitioners must learn not only to recognize shock, but also to evaluate nutritional status and perform frequent patient reassessments to guide fluid management. Although there are several published simulation-based curricula focused on hypovolemic shock in pediatric patients [2, 32, 33], there are few publications evaluating curricula focused on shock of any kind in pediatric patients with co-morbid malnutrition in RLS [34, 35].

In our study, we used medium-fidelity mannequin-based simulation as part of an educational program designed to teach Filipino pediatric residents shock concepts, with an emphasis on fluid resuscitation, nutritional status assessment, and patient re-evaluation. We hypothesized that implementing a hands-on shock curriculum would decrease time to fluid administration on a simulated mannequin. In addition, we hypothesized that this curriculum would improve comfort and knowledge regarding shock recognition and fluid management based on varying nutritional status. Improving recognition of the shock state, combined with cautious initiation of fluids and improved patient assessment skills, could be lifesaving and prevent mortality of children suffering from various forms of shock.

## Materials/methods

**Fig. 1:**
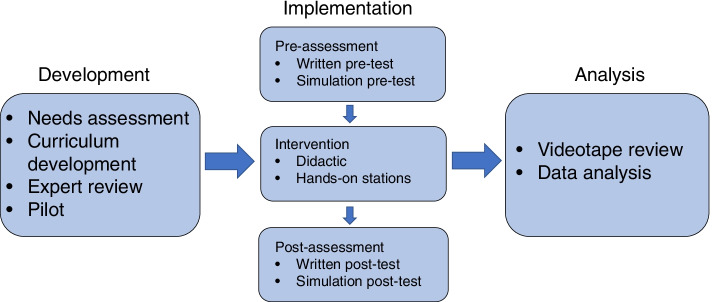
Study design

In this pre/post prospective cohort study, we evaluated a shock curriculum implemented over the course of a half-day workshop at Philippine Children’s Medical Center (PCMC) (Fig. 1**)**, a tertiary care pediatric center in Manila. Ethics approval was obtained through Johns Hopkins University School of Medicine (JHUSOM) institutional review board, in addition to the PCMC office of research development. PCMC is one of the more resourced pediatric hospitals in the Philippines. The hospital has pediatric specialists, intensive care units with some limited ventilator capacity, a dialysis unit and access to some imaging studies such as simple CT scan and ultrasound. However, resource limitations still exist regarding laboratory frequency/availability, number of and access to mechanical ventilation, lack of supplies for central venous access, and the like, making the setting vastly different from a tertiary care pediatric facility in many other settings. In addition, the residents who train at PCMC will ultimately work in different settings across the country and may need to practice independently with minimal pediatric-specific resources. For this reason, we chose pediatric residents rotating at PCMC as participants for our pilot study. The intervention was implemented in March 2020 with 24 pediatric residents. Simulation cases were piloted and all materials, including cases, questionnaires, checklists, and didactics, were reviewed for content and fidelity by pediatric critical care fellows and attending physicians at both PCMC and JHUSOM. The residents were randomly divided between a morning and an afternoon session to ensure instructor to participant ratio of 4:1. We made no modifications to any materials between sessions to effectively compare the two cohorts.

Participants were given a written pre-test, which included questions about demographic information, an assessment of confidence with shock management on a 5-point Likert scale, and 10 knowledge-based multiple-choice questions on shock concepts. Six of these questions pertained to shock concepts typically taught as part of Pediatric Advanced Life Support (PALS) course content. Four additional questions were added on subjects of IO access, response to resuscitation, malnutrition, and management of shock secondary to dengue hemorrhagic fever. The questions underwent content review by a total of eight pediatric critical care fellows and attendings. We also administered the questions to non-curriculum participants at various levels of training as part of this process to assess discriminatory validity of the written questions. After the written exam, each resident participated individually in a simulation-based objective standardized clinical examination (OSCE) using a medium-fidelity mannequin that presented in hypovolemic shock with co-morbid malnourished status. Due to the inability to connect the mannequin to the simulation control box, we used the mannequin in a low-fidelity mode. Therefore, we were unable to change vital signs and physical exam findings in response to participant interventions. Mannequin capabilities included static palpable pulse, breath sounds, and ability to place IO. The simulation facilitator provided the participant with the clinical vignette background/history. In order to mimic a RLS, supplies required for the OSCE included intravenous/IO supplies, fluids, and the ability to check glucose. Sessions were videotaped and scored in real time by the primary investigator, who used a checklist to evaluate for the primary outcome, time in seconds to initiation of fluid resuscitation. This checklist was not formally validated. Content and construct of the checklist was modeled on previously validated checklists for pediatric resuscitation scenarios, identified by Medline literature review of simulation-based pediatric resuscitation programs [5, 8, 10]. These checklists were compiled and modified to reflect skills performed in the OSCE. Additionally, the checklist was reviewed by eight pediatric critical care specialists, four from each institution. Items on the checklist included oxygen delivery, appropriate choice and administration of fluid, assessment for malnutrition, capillary refill assessment, correct placement of IO access, identification and appropriate treatment of hypoglycemia, and reassessment of physical exam and vital signs between fluid boluses (Additional file 1: Appendix). Videotaping of each scenario allowed for later re-review by the primary investigator to confirm intra-rater reliability. Two additional reviewers blinded to pre/post assessment were trained and reviewed 20% of the videos to ensure inter-rater reliability.

The intervention included a didactic curriculum taught by the primary investigator. Topics included shock definitions, presenting symptoms, fluid types, and management recommendations guided by the Surviving Sepsis Campaign, with a differentiation based on nutritional status and concern for sepsis or dengue hemorrhagic fever. The didactic session was followed by three hands-on skills stations covering (1) oxygen delivery, assisted ventilation, and patient reassessment; (2) emergency access and rapid fluid initiation/administration; and (3) dextrose administration for hypoglycemia, appropriate assessment/evaluation for malnutrition, and importance of timely antibiotic administration in some forms of shock. Each skills station was taught in small groups by a pediatric intensive care fellow or attending physician. After the intervention, participants repeated the same written test as they had completed at the beginning of the session. Each participant again attempted a videotaped OSCE as an individual. The post-intervention scenario utilized a different clinical vignette stem to minimize recall bias, but was similarly a malnourished patient presenting in hypovolemic shock who required an identical series of interventions to achieve successful resuscitation from shock**.** In addition to the primary outcome of time to initiation of fluids, secondary outcomes included participant self-reported improvement in confidence with shock concepts, participant improvement in knowledge of shock concepts measured on the written post-test, and total score on the OSCE checklist.

### Statistical analysis

To achieve 80% power and an α level of 0.05 to detect an improvement of 1 minute in performance, we calculated that we would need 21 training participants for target enrollment. Analysis was carried out with Stata/IC 16.1 (StataCorp). Outcome measures were compared before and after curriculum implementation. Medians with interquartile ranges (IQR) were reported for nonparametric data. Each pairing of variables/outcome measures was compared by Wilcoxon Sign Rank test. Statistical significance was set at *p* < 0.05. Subgroup analysis by cohort was performed using χ [2] and by resident postgraduate year (1, 2, or 3) using Kruskal-Wallis. Sub-item performance on the checklist was evaluated by using McNemar’s paired proportion. Spearman correlation was used for intra-rater and inter-rater reliability with a goal of *ρ* > 0.8.

## Results

As a result of the COVID-19 pandemic, enrollment in the research study was stopped prematurely when the Philippines shut its borders to non-citizens and PCMC forbade gatherings of more than 10 people per room. A total of 24 pediatric residents were enrolled prior to this shutdown (Table 1). Of these participants, 96% were female, 71% had participated in simulation 1–3 times, and all were certified in Basic Life Support (BLS) and PALS. Nine participants were post-graduate year (PGY) 1, seven were PGY-2 and eight were PGY-3.

**Table 1:** Demographics of residents who participated in the simulation-based training (*n* = 24)

Characteristic	N (%)
Female	23 (96)
Resident year	
PGY 1	9 (38)
PGY 2	7 (29)
PGY 3	8 (33)
Simulation experiences	
0	4 (17)
1–3	17 (71)
> 4	3 (13)
BLS/PALS trained	24 (100)

*Abbreviations*: *BLS* Basic Life Support; *PALS* Pediatric Advanced Life Support; *PGY* Postgraduate year

Individuals reported a median of 4 out of 5 on a Likert scale in regard to their confidence in English in the medical setting (IQR 4–4). Despite median confidence with some shock concepts remaining the same post-intervention, on analysis, confidence in all domains improved significantly (*p* < 0.01; Table 2). Test scores showed no significant improvement post-intervention (Table 2). The time to initiation of fluids showed a non-significant decrease from 71.5 seconds to 68 seconds, but the total score on the checklist improved (*p* < 0.01; Table 2).

**Table 2:** Comparison of self-reported confidence, written test scores, and simulation OSCE performance before and after the intervention (*n* = 24)

Parameter	Pre-Intervention Median (IQR)	Post-Intervention Median (IQR)	***p*** Value*
Self-reported confidence^a^			
Identification of shock	4 (3–4)	4 (4–5)	**< 0.01**
Identification of malnutrition	3 (3–4)	4 (4–4)	**< 0.01**
Management of hypovolemic shock	4 (3–4)	4 (4–5)	**< 0.01**
Management of septic shock	3 (3–4)	4 (4–4)	**< 0.01**
Placement of an IO needle	3 (2–3.5)	4 (4–4)	**< 0.01**
Written knowledge test score^b^			
Overall	10 (9.5–10)	10 (10–10)	0.15
PGY-1	10 (10–10)	10 (10–10)	
PGY-2	10 (9–10)	10 (10–10)	
PGY-3	10 (9–10)	10 (9–10)	
Time to initiation of fluid in simulation OSCE (seconds)			
Overall	71.5 (52–116.5)	68 (52.5–89)	0.42
PGY-1	97 (64–123)	84 (49–95)	
PGY-2	63 (49–90)	68 (68,90)	
PGY-3	71.5 (52.5–114.5)	57.5 (52.5–73.5)	
Checklist total in simulation OSCE			
Overall	10 (8.5–11)	15 (13–16)	**< 0.01**
PGY-1	10 (9–12)	15 (13–16)	
PGY-2	9 (8–10)	15 (14–16)	
PGY-3	10.5 (8.5–11.5)	15 (12.5–16.5)	

*Abbreviations*: *IO* Intraosseous; *IQR* Interquartile range; *PGY* Postgraduate year; *OSCE* Objective standardized clinical examination

*Comparisons were made by Wilcoxon sign rank. Bold font indicates statistical significance

^a^Rated on a 5-point Likert scale, where 1 = no confidence and 5 = extremely confident

^b^Maximum score on written test was 10

No differences were apparent by postgraduate year on sub-analysis for any outcome. Significant improvement on the checklist was seen in skills related to appropriate malnutrition assessment, initiation of oxygen therapy, choice of correct isotonic fluid for volume resuscitation, correct placement of the IO needle, correct reassessment of patient post-interventions, and identification and treatment of hypoglycemia (Table 3).

**Table 3:** Number of participants who successfully completed each sub-item on simulation scenario OSCE checklist pre- and post-intervention (*n* = 24)

Sub-item	Pre-Intervention^a^ n (%)	Post-Intervention^a^ n (%)	Mean Difference (95% CI)	***p*** Value†
Checks VS	24 (100)	24 (100)	0 (− 0.04 to 0.04)	1
Includes BP, RR, SPO_2_, HR	7 (29)	7 (29)	0 (− 0.2 to 0.2)	1
Assesses for malnutrition	4 (17)	18 (75)	−0.58 (− 0.85 to − 0.31)	**< 0.01**
Assesses airway	12 (50)	20 (83)	− 0.33 (− 0.65 to − 0.02)	0.06
Assesses breathing	7 (29)	8 (33)	− 0.04 (− 0.22 to 0.14)	1
Places O_2_	11 (46)	23 (96)	−0.5 (− 0.77 to − 0.23)	**< 0.01**
Administers O_2_ correctly	11 (46)	23 (96)	−0.5 (− 0.77 to − 0.23)	**< 0.01**
Checks pulse	17 (71)	12 (50)	0.21 (− 0.03 to 0.45)	0.13
Pulse central	1 (4)	0 (0)	0.04 (−0.08 to 0.16)	1
Checks capillary refill	8 (33)	20 (83)	−0.08 (− 0.35 to 0.19)	0.73
Initiates fluids within 5 min	24 (100)	24 (100)	0 (−0.04 to 0.04)	1
Correct fluid choice	0 (0)	13 (54)	−0.58 (− 0.82 to − 0.34)	**< 0.01**
Attempts IO needle placement	24 (100)	24 (100)	0 (−0.04 to 0.04)	1
Places IO correctly	8 (33)	23 (96)	−0.63 (− 0.86 to − 0.39)	**< 0.01**
Rechecks VS	24 (100)	22 (92)	0.08 (−0.07 to 0.24)	0.5
Includes BP, RR, SPO_2_, HR	6 (25)	5 (21)	0.04 (−0.14 to 0.22)	1
Reassesses patient	17 (71)	21 (88)	−0.17 (− 0.4 to 0.06)	0.22
Includes lung auscultation and palpation of liver edge in reassessment	0 (0)	11 (46)	−0.46 (− 0.7 to − 0.22)	**< 0.01**
Checks glucose	11 (46)	23 (96)	−0.5 (− 0.74 to − 0.26)	**< 0.01**
Administers dextrose	11 (46)	21 (88)	− 0.42 (− 0.66 to − 0.18)	**< 0.01**
Correct dextrose dose	10 (42)	21 (88)	−0.46 (− 0.7 to − 0.22)	**< 0.01**

*Abbreviations*: *BP* Blood pressure; *CI* Confidence interval; *HR* Heart rate; *IO* Intraosseous; *O*_*2*_ Oxygen; *RR* Respiratory rate; *SPO*_*2*_ Oxygen saturation by pulse oximetry; VS Vital signs

^a^*n* = 24 both pre- and post-intervention

†Comparisons were made by McNemar’s paired proportions. Bold font indicates statistical significance

Intra-rater agreement was good for the total checklist score (ρ = 0.97; *p* < 0.001; mean difference = 0.06; 95% confidence interval [CI], − 0.18-0.31) and time to initiation of fluid (*ρ* = 0.98; *p* < 0.001; mean difference = 3.3; 95% CI, 1.8–4.9). Similarly, inter-rater reliability was good for the total checklist score (*ρ* = 0.81; *p* = 0.004; mean difference = 1.3; 95% CI, 0.4–2.2) and time to initiation of fluid (*ρ* = 0.98; *p* < 0.001; mean difference = 0.6; 95% CI, − 3.5-2.38).

## Discussion

Despite universal evidence that early recognition of shock and goal-directed therapy with fluids is beneficial, rapid high-volume fluid resuscitation may not be the correct management in all populations [17]. The WHO and the Surviving Sepsis Campaign differentiate management based on critical care availability, and stress judicious fluid resuscitation and frequent reassessments with particular attention to signs of fluid overload [16, 18]. We were successful in designing, implementing, and studying a simulation-based educational program that focused on immediate recognition of shock, obtaining access, examining the patient and identifying signs of malnutrition, carefully choosing fluids, and frequently reassessing patients for response to therapy or signs of fluid overload.

Following our educational intervention, the participants showed no significant improvement in time to initiation of fluids. However, all residents started fluids within 5 minutes of case start both pre- and post-intervention, consistent with the gold standard for fluid initiation in shock. Scores on the written knowledge-based test did not improve, which may be due to the high median pre-test scores or to the limited number of questions. Both of these findings may also be due to the participants being previously trained in and knowledgeable about PALS algorithms. Similar to prior studies, participants showed consistent improvement in confidence with shock resuscitation skills [2, 3]. Additionally, total checklist scores increased post-intervention. Residents showed improvement in recognition of malnutrition, placement of the patient on oxygen, choice of correct fluid, administration of fluid at an appropriate rate, correct placement of an IO needle, reassessment of the patient for fluid overload, and evaluation and treatment of hypoglycemia. Interestingly, these represent the skills taught in the hands-on stations. The primary outcome of time to initiation of fluids is likely a poor single marker of good clinical care. The rapidity with which the residents started a fluid bolus often precluded a full assessment and reassessment of the patient. Therefore, the majority of participants were unable to identify a malnourished patient, recognize decompensation, and modify management on the pre-test OSCE. Although administration of fluids in a timely manner is essential, accurate choice of fluids, ability to place alternative emergency access, and patient reassessment skills are likely all equally important, skills represented by total score on the checklist.

Similar to checklist use in other simulation studies on shock [5–8, 10], the total score on the checklist may be a better representation of knowledge acquisition than the written test and a more clinically relevant outcome than simply time to initiation of fluids. Although the checklist is not formally validated, we did complete components of the validation process, including partial expert review and assessment for intra- and inter-rater reliability. Checklist content was reviewed by senior pediatric intensive care specialists from both the United States and the Philippines, and we showed good intra- and inter-rater agreement on analysis. While the resuscitation checklist still needs to undergo a formal validation study, it could potentially be applied to clinical scenarios as a marker of change in practice and management in the future.

Our study had several limitations. It was a small pilot study to evaluate feasibility of curriculum implementation in this setting, made smaller by international circumstances during a pandemic. Additionally, our power calculation overestimated resident time to initiation of fluids and the checklist we used to evaluate our outcomes is not yet a validated tool. PCMC is a large academic institution with residents who are BLS- and PALS-certified, generally experienced in simulation, and comfortable speaking English in medically complex situations. Although reflective of the typical make-up of pediatric trainees, participants were almost exclusively female. The power and generalizability of our study are therefore limited. It would be beneficial to continue implementation with more residents and to extend the curriculum to healthcare workers from various regions of the country. Although not the case in our study, lack of familiarity with simulation is a common limitation in RLS, emphasizing the importance of introducing the mannequin functionality and the concept of simulation and debriefing. Importantly, this curriculum is not fully applicable to some rural and low-resource areas, as the simulation minimally requires access to intravenous/IO supplies, fluids, and the ability to check glucose. In these lowest resource areas, the curriculum could be adapted to focus on physical exam skills and early recognition of shock to guide healthcare workers in patient triage and escalation to a higher level of care. Additionally, we used a medium-fidelity mannequin for the OSCE, which is an expensive tool. However, we experienced technical challenges with the mannequin, essentially rendering it a low-fidelity mannequin. The OSCE could easily be modified for implementation using a low-fidelity mannequin. Finally, although residents showed improvement in confidence and knowledge as measured by the checklist, it is unclear how our results will translate both long-term, and to the clinical setting. As a future direction of this study, it will be important to assess skill and long-term knowledge retention at various intervals following this curriculum. Participants would likely benefit from refresher training using a low-dose/high-frequency training paradigm that would help them to maintain their skills and facilitate translation of their knowledge into improved clinical practice [5, 16].

This educational program included a simulation portion, a didactic and multiple hands-on stations. Particularly in a setting where time, materials and personnel are limited, it would be helpful to know if all components of this program are essential for knowledge acquisition. Future iterations of the study could compare outcomes between individuals who participated in the entire curriculum, individuals who participated solely in the didactic and those who did not participate in any intervention. This could help delineate if one intervention is more effective than another, as it is possible that the OSCE on its own serves as the most substantial contributor to participant learning.

Global health research poses many unique challenges. We had a previously established relationship with individuals practicing pediatric critical care at PCMC [36], which enabled us to implement this curriculum more efficiently than may be typical. Despite this strong relationship with individuals at PCMC, the formal approval process for the project took several months, necessitating in-person meetings with hospital administrators and the establishment of an in-country proxy to attend additional meetings, obtain signatures and deliver paperwork. Remote communication and collaboration on educational materials was especially difficult. This improved significantly following our initial site visit, where we were able to better understand which channels of communication (e.g. phone messenger applications) were most utilized by our colleagues. Even with these positive interactions, we were unable to obtain approval to enroll non-physicians, and were limited in the dates for the experience due to difficulties finding protected time for the residents. However, in part due to excellent communication and preparation (materials, logistics, staffing), the day of implementation was very successful. Students expressed gratitude for the experience, requested to stay after their sessions were complete to ask questions and practice skills, and indicated that they would be very interested in future hands-on simulation experiences.

## Conclusions

This study of a simulation-based education program on shock for pediatric residents in Manila was designed to establish a sustainable model of simulation education using accessible, low-cost materials in an RLS. All materials that we used, with the exception of the medium fidelity mannequin, were donated to PCMC in order to promote sustainability of the curriculum and refresher training sessions. We showed improvement with respect to confidence in skills needed for resuscitation of children in shock, in addition to knowledge as measured by a resuscitation OSCE checklist after our intervention. Based on the results of this study and the success of the curriculum, PCMC plans to use local faculty to continue teaching this curriculum to its pediatric residents, a practice that will enhance project sustainability.

We were able to successfully collaborate with local physicians who participated in the creation and implementation of the curriculum. We have laid the groundwork for continued simulation education at PCMC that could be elaborated upon to encompass other topics and scenarios beyond shock in a contextualized manner. If successful in creating a larger and more long-term educational network, over time we plan to evaluate for changes in clinical practice, and eventual changes in patient outcomes, with the intent to improve recognition and management of shock and decrease the associated mortality. Our results are promising, but transitioning from improvement in confidence and knowledge to changes in clinical outcome will require widespread and long-term use of an educational program that comes from local stakeholder commitment and institutional buy-in.

## Supplementary Information

**Additional file 1.**

## Data Availability

Data and materials for implementing curriculum have been uploaded and are available for public access in Harvard Dataverse, V2. https://dataverse.harvard.edu/dataset.xhtml?persistentId=doi:10.7910/DVN/OQEMLH

## Abbreviations

RLS: Resource-limited settings
IO: Intraosseous
WHO: World Health Organization
FEAST: Fluid Expansion as Supportive Therapy
PCMC: Philippine Children’s Medical Center
JHH: Johns Hopkins Hospital
PALS: Pediatric Advanced Life Support
OSCE: Objective standardized clinical examination
BLS: Basic Life Support
PGY: Post-graduate year
