# Supplementary material for: Implementation and evaluation of a shock curriculum using simulation in Manila, Philippines: a prospective cohort study
Source: BMC Med Educ. 2022 Aug 5;22:606. doi: 10.1186/s12909-022-03669-0 (PMC9354294; doi:10.1186/s12909-022-03669-0)
Supplement: Supplementary file 1 — Additional file 1. [file 12909_2022_3669_MOESM1_ESM.docx]

**Additional file 1:**

**Skills stations:**

Each station taught by a pediatric critical care fellow or attending. Groups of 3-5 rotate through each 20-minute station.

**Station 1:** Oxygen delivery, assisted ventilation, and patient assessment

Materials needed:

- NeoNatalie mannequin (or similar low fidelity simulation mannequin)
- Computer with sound
- Laerdal Sim Junior (or similar medium fidelity simulation mannequin)
- Oxygen delivery and ventilation systems: low-flow nasal cannula, simple facemask, non-rebreather mask and bag-valve mask
- Oral airway, nasal airway
- Playdough (to create simulated hepatomegaly within NeoNatalie mannequin)

Skills taught:

- Patient assessment starting with Airway, Breathing, Circulation (ABCs)
- Describe indications for various oxygen delivery devices and show examples of the following: low-flow nasal cannula, heated high flow, simple facemask, Venturi facemask, non-rebreather, CPAP/BIPAP
  - Video resource
    - Simulation Use for Global Away Rotations PEARLS. The Board of Regents of the University of Wisconsin System (SUGAR Project) 2021. Oxygen Delivery Devices. Accessed August 21, 2021. <https://sugarprep.org/videos/#Oxygen_Delivery_Devices>
- Ventilation using Bag-valve mask on NeoNatalie, including appropriate mask fit and how to troubleshoot ineffective ventilation (reposition, two-person technique, oral airway)
- Demonstrate pediatric physical assessment (vital signs, auscultation of heart and lungs, abdominal exam, pulses and perfusion) and importance of reassessment including evaluation for fluid overload (crackles, gallop, hepatomegaly) – can demonstrate on medium-fidelity mannequin and capillary refill time on each other, hepatomegaly (playdough in RUQ of abdomen underneath skin covering of Laerdal Sim Junior and NeoNatalie to mimic congestive liver enlargement with fluid overload)
  - Crackles (video clip)
    - Medzcool. Fine Crackles (Rales) – Lung Sounds. YouTube. Accessed February 25, 2020. <https://youtu.be/LHqqvrm2j6g>
  - Gallop (video clip)
    - Thinklabs. S4 Gallop – Normal Speed. YouTube. Published January 28, 2016. Accessed February 25, 2020. <https://www.youtube.com/watch?v=lgHkK1x5kmw>
    - Medzcool. S4 Heart Sound. YouTube. Accessed February 25, 2020. <https://www.youtube.com/watch?v=KcMF8rJDTIk>
  - Capillary refill time (video clip)
    - Dr. Borst’s Occupational Therapy Classroom. Capillary Refill Test. YouTube. Published February 5, 2019. Accessed February 25, 2020. <https://www.youtube.com/watch?v=n--wFoZFklg>

**Station 2:** Emergency access and rapid fluid initiation/administration

Materials needed:

- IV pole with several bags of Isotonic intravenous fluid
- Bucket
- Three-way stop-cock
- Manual Intraosseous (IO) needles
- Tape
- Alcohol pads
- IV extension tubing
- Syringe
- Saline Flush
- 18-gauge IV catheter/ needle and/or adult lumbar puncture needle
- IO leg partial task trainer (or mannequin with capability to place IO access)

Skills taught:

- Demonstrate correct placement of IO needle using manual technique with manual IO and 18 gauge needle or adult lumbar puncture needle
  - Video resource
    - Simulation Use for Global Away Rotations PEARLS. The Board of Regents of the University of Wisconsin System (SUGAR Project) 2021. Intraosseous Needle Placement. Accessed August 21, 2021. <https://sugarprep.org/videos/#Intraosseous_Needle_Placement>
- Demonstrate rapidity of push-pull of fluid using syringe method and three-way stopcock with different sized needles into bucket

**Station 3:** Dextrose administration for hypoglycemia, appropriate assessment/evaluation for malnutrition, and importance of timely antibiotic administration in some forms of shock

Materials needed:

- NeoNatalie mannequin (or similar low fidelity simulation mannequin)
- Glucometer
- SAFingerStick (JCCC Foundation) glucose finger stick simulator https://www.jccc.edu/student-resources/healthcaresimulation/jc3innovations/safingerstick.html
- Red food coloring and dextrose of various concentrations (5%, 10%, 25%, plus sterile water)
- Mid upper arm circumference (MUAC) tape

Skills taught:

- Evaluate for malnutrition using (MUAC) tape on NeoNatalie using example case below
  - Video resource
    - Rohatgi, S. How to measure mid upper arm circumference (MUAC) – Kalawati Saran Children’s Hospital, New Delhi. YouTube. Published June 23, 2017. Accessed August 21, 2021. <https://www.youtube.com/watch?v=uQb8fge-BWs>
- Demonstrate calculation of pediatric early warning score (PEWS) using example case below
- Demonstrate appropriate use of glucometer and calculate correct dose of dextrose, including practicing dilution using example case below
- Identify when to consider antibiotics in shock

You are caring for a two-year-old girl on the wards who was admitted with pneumonia. She is now on 2 Liters Nasal Cannula, 100% FiO2 with the following vital signs and physical exam:

HR: 140 BPM BP: 78/45 mmHg RR: 54 SpO2: 95% Wt: 20 kg

Neuro: Patient is sleeping

CV: Capillary refill 3 seconds

Respiratory: Nasal flaring, crackles in RLL

What is her PEWS score (**Figure 1/Table 1**)?

Figure 1: Normal vital signs for age


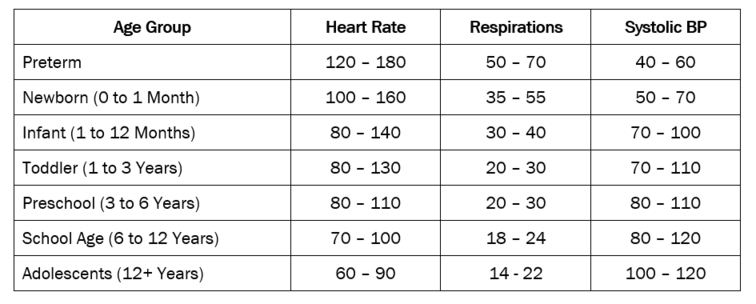


Abbreviations: BP blood pressure

Table 1: Pediatric early warning scoring system

|  | 0 | 1 | 2 | 3 |
| --- | --- | --- | --- | --- |
| Neuro | Appropriate | Sleeping | Irritable | Lethargic/confused OR reduced response to pain |
| CV | Pink OR Capillary refill 1-2 seconds | Pale OR Capillary refill 3 seconds | Gray OR capillary refill 4 seconds  Tachycardia >20 above normal rate | Gray/mottled, capillary refill >/=5,  Tachycardia >30 above normal OR bradycardia |
| Respiratory | Within normal parameters | RR > 10 above normal, mild increase in WOB | RR > 20 above normal, moderate increase in WOB | Five below-normal parameters with severely increased WOB (retractions, grunting) |

Abbreviations: CV cardiovascular, RR respiratory rate, WOB work of breathing

Check her glucose.

Her glucose is 2.4 mmol/dL (43 g/dL). You have 10% dextrose solution. Calculate how much dextrose solution you should administer to this patient.

Rule of 50s:

D5% - give 10 cc/kg

D10% - give 5 cc/kg

D25% (must be central) – give 2 cc/kg

D50% (must be central) – give 1 cc/kg

What if you only have 50% dextrose solution? 25% dextrose solution? How can you dilute it to 10% dextrose?

Assess the mannequin for malnutrition using a mean upper arm circumference

**Simulation case scenarios (Tables 2 and 3), checklist (Table 4) and debriefing guide (Table 5)**

**Materials:**

- - Low or medium fidelity mannequin with IV in place, including mannequin adaptation to mimic patient hepatomegaly (we placed Playdough under the skin-covering in the right upper quadrant of the mannequin’s abdomen to simulate hepatomegaly) and ability to place IO
  - Stethoscope
  - Oxygen delivery source (nasal cannula, simple facemask or non-rebreather)
  - Mid upper arm circumference (MUAC) tape used to assess nutritional status
  - Bags of fluid (lactated Ringer’s OR 0.9% sodium chloride; dextrose 10%; dextrose 5% in 0.45% sodium chloride OR dextrose 5% in lactated Ringer’s; oral rehydration solution)
  - Alcohol pads and gloves
  - Manual intraossesous needle with saline flush, empty syringe, tubing and tape for stabilization
  - Glucose monitor
  - Nasogastric tube with lubricant for mannequin

Table 2: Scenario script – Case one

| **White = Background for Leaders** | | **Light Gray – Read Script Aloud** | **Dark Gray = Read if asked/needed** |
| --- | --- | --- | --- |
| **Learning Goals** | Rapid assessment and management of a patient with hypovolemic shock and malnutrition   - Recognize shock and likely cause (diarrheal illness) - Obtain timely and frequent vital signs and assessments - Evaluate for and identify malnutrition - Initiate therapeutic interventions specifically rapid IV/IO access, administration of fluids appropriate for a malnourished patient, and identify and correct hypoglycemia | | |
| **Supplies** | - Laerdal Sim Junior mannequin functioning in a low fidelity mode (will be described as a young child) - IV is in place, IO supplies, NG supplies, fluids, syringes, oxygen, glucose monitor | | |
| **Before Starting** | - Include 10 minutes orienting participants to mannequin, supplies and the concept of simulation - **“**Remember, treat this case like you would an actual case.” - **“**If you need information from the patient’s exam, perform the exam and ask for the findings.” - “We will let you know when the case is done. Are there any questions?” | | |
| **Case Briefing** | “You are called to the bedside to evaluate a four-year-old male who came in yesterday with diarrhea and vomiting. He is no longer responding to questions like he was before. You have access to a nurse (played by the leaders) who can answer questions, provide vital signs and describe how and what the patient is doing. Assume you have access to any supplies, medications, labs you would have in the hospital.” | | |
| **Narrative Description** | “Help. This patient seems sleepier than he was before.”  *IF ASKED: No PMH; malnourished; HIV neg; no fever, watery diarrhea 6 times in last hour, no blood in stool, 2 episodes of non-bloody/non-bilious emesis, unable to drink anything* | | |
| **Initial Vital Signs** | *Provide each if asked for that specific vital sign*  Wt. 10 kg T 37.6 HR 160 RR 30 BP 65/35 O2 90% Z-score -3.2 MUAC 110 mm | | |
| **Initial Exam** | *Provide exam findings if asked for them specifically (i.e. “What is his abdominal exam?”),*  GEN: Responds to pain, moans when asked a question  HEENT: Pupils equal, round and reactive; lips dry, supple neck, sunken eyes  CVS/RESP: Tachycardic with no murmur; lungs clear to auscultation  ABD: Abdomen soft, mild diffuse tenderness, no rebound, hyperactive bowel sounds no hepatosplenomegaly  EXT: Capillary refill 5 seconds, hands cool to touch, pulses weak and fast, skin remains tented when pinched, bilateral edema of feet and muscle wasting | | |
| **Vital Signs/Exam Changes & Labs** | - Admission hemoglobin = 8 g/dL - IV will stop working after fluid resuscitation is initiated and unable to place another one - If asked for repeat vitals/exam findings, adjust accordingly based on interventions: If D5 ½ NS or D5 LR is initiated over an hour, HR and BP improve, if crystalloid is given quickly, patient will get more tachycardic, develop crackles and hepatomegaly - Blood glucose 2.4 mmol/L (43 g/dL) -> (5.3 mmol/L or 95 g/dL if dextrose bolus given) | | |
| **Ideal Interventions**  **w/ Possible Rescue Sentences** | - ABCs; apply oxygen therapy - Recognize hypovolemic shock and treat appropriately:   - Determine that patient is malnourished  - Identify and treat hypoglycemia (5cc/kg of D10 solution)  - Initiate fluids (15cc/kg D5 1/2NS or D5LR over 1 hour) (*what kind of fluid should we give? How fast should we give the bolus?)*  *-*When IV fails, place IO and continue resuscitation   - Alternative: Place NG, give ReSoMal fluid at 5cc/kg every 30 minutes - *Once fluid initiated via IO “It has been 15 minutes since you initiated fluids. Is there anything else you would like to do for this patient?” OR if no NG or IO placed, within 2 minutes of failed IV “It has been 15 minutes since the patient received fluids, is there anything else…”* - Ask for repeat vital signs and repeat physical exam (listen to lungs and heart, feel for liver, CRT) - Obtain blood tests (RBG, CBC, BMP and blood culture if possible) | | |
| **Simulation Operation During Scenario** | **Team Intervention Simulator Effect**   1. Identify/treat hypovolemic shock, HR decreases, blood pressure improves   in malnourished patient Mental status improves   1. Identify/treat hypoglycemia Glucose improves | | |

Abbreviations: IV intravenous, IO intraosseous, NG nasogastric, PMH Past medical history, WT weight, T temperature, HR heart rate, RR respiratory rate, BP blood pressure, O2 oxygen, MUAC mid upper arm circumference, GEN general, HEENT head eyes ears nose throat, CVS/RESP cardiovascular system/respiratory, ABD abdominal, EXT extremities, D5 dextrose 5%, NS normal saline, LR lactated ringers, ABCs, airway breathing circulation, RBG rapid blood glucose, CBC complete blood count, BMP basic metabolic panel

Table 3: Scenario script – Case two

| **White = Background for Leaders** | | **Light Gray – Read Script Aloud** | **Dark Gray = Read if asked/needed** |
| --- | --- | --- | --- |
| **Learning Goals** | Rapid assessment and management of a patient with hypovolemic shock and malnutrition   - Recognize shock and likely cause (diarrheal illness) - Obtain timely and frequent vital signs and assessments - Evaluate for and identify malnutrition - Initiate therapeutic interventions specifically rapid IV/IO access, administration of fluids appropriate for a malnourished patient and identify and correct hypoglycemia | | |
| **Supplies** | - Laerdal Sim Junior mannequin functioning in a low fidelity mode (will be described as a young child) - IV in place, IO supplies, NG supplies, fluids, syringes, oxygen, glucose monitor | | |
| **Before Starting** | - Include 10 minutes orienting participants to mannequin, supplies and the concept of simulation - **“**Remember, treat this case like you would an actual case.” - **“**If you need information from the patient’s exam, perform the exam and ask for the findings.” - “We will let you know when the case is done. Are there any questions?” | | |
| **Case Briefing** | “You are called to the bedside to evaluate a six-year-old male who presented today with abdominal pain and diarrhea. He is no longer drinking anything. You have access to a nurse (played by the leaders) who can answer questions, provide vital signs and describe how and what the patient is doing. Assume you have access to any supplies, medications, labs you would have in the hospital.” | | |
| **Narrative Description** | “Help. I’m worried about this patient. He has not been drinking.”  *IF ASKED: No PMH; malnourished; HIV neg; no fever, watery diarrhea once every hour, refusing to drink, last oral intake was yesterday, which he vomited. No blood in stool.* | | |
| **Initial Vital Signs** | *Provide each if asked for that specific vital sign*  Wt. 15 kg T 37.6 HR 150 RR 25 BP 72/30 O2 90% Z-score -3 MUAC 110 mm | | |
| **Initial Exam** | *Provide exam findings if asked for them specifically (i.e. “What is his abdominal exam?”),*  GEN: Responds to pain, moans when asked a question  HEENT: Pupils equal, round and reactive; lips dry, supple neck, sunken eyes  CVS/RESP: Tachycardic with no murmur; lungs clear to auscultation  ABD: Abdomen soft, diffuse mild tenderness, no rebound, hyperactive bowel sounds no hepatosplenomegaly  EXT: Capillary refill 6 seconds, hands are cool to touch, pulses weak and fast, skin remains tented when pinched, bilateral edema of feet and muscle wasting | | |
| **Vital Signs/Exam Changes & Labs** | - Admission hemoglobin = 8 g/dL - IV will fail after initiation of fluids and unable to place another one - If asked for repeat vitals/exam findings, adjust accordingly based on interventions: If D5 ½ NS or D5 LR is initiated over an hour, HR and BP improve, if crystalloid is given quickly, patient will get more tachycardic, develop crackles and hepatomegaly - Blood glucose 2.2 mmol/L (37 g/dL) -> (5.5 mmol/L or 100 g/dL if dextrose bolus given) | | |
| **Ideal Interventions**  **w/ Possible Rescue Sentences** | - ABCs; apply oxygen therapy - Recognize hypovolemic shock and treat appropriately:   - Determine that patient is malnourished  - Identify hypoglycemia (5cc/kg of D10 solution)  - Initiate fluids (15cc/kg D5 1/2NS or D5LR over 1 hour) (*what kind of fluid should we give? How fast should we give the bolus?)*  - When IV fails, place IO and continue resuscitation   - Alternative: Place NG, give ReSoMal fluid at 5cc/kg every 30 minutes - *Once fluid initiated via IO “It has been 15 minutes since you initiated fluids. Is there anything else you would like to do for this patient?” OR if no NG or IO placed, within 2 minutes of failed IV “It has been 15 minutes since the patient received fluids, is there anything else…”* - Ask for repeat vital signs and repeat physical exam (listen to lungs and heart, feel for liver, CRT) - Obtain blood tests (RBG, CBC, BMP and blood culture if possible) | | |
| **Simulation Operation During Scenario** | **Team Intervention Simulator Effect**   1. Identify/treat hypovolemic shock, HR decreases, blood pressure improves   in malnourished patient Mental status improves   1. Identify/treat hypoglycemia Glucose improves | | |

Abbreviations: IV intravenous, IO intraosseous, NG nasogastric, PMH Past medical history, WT weight, T temperature, HR heart rate, RR respiratory rate, BP blood pressure, O2 oxygen, MUAC mid upper arm circumference, GEN general, HEENT head eyes ears nose throat, CVS/RESP cardiovascular system/respiratory, ABD abdominal, EXT extremities, D5 dextrose 5%, NS normal saline, LR lactated ringers, ABCs, airway breathing circulation, RBG rapid blood glucose, CBC complete blood count, BMP basic metabolic panel

Case timeline for leaders:

Time 0 = After case scenario reading is complete

Time to initiation of fluids = When participant verbalizes what kind of fluid to give

IV fails after fluid initiation and unable to place another IV “You are unable to place another IV”

After IO or NG placed with fluid administration, time will progress 15 minutes “It has been 15 minutes since fluid initiation, is there anything else you would like to do for this patient?”

If participant does not attempt to place IO or NG within 2 minutes of IV failure, progress case 15 minutes “It has been 15 minutes since the patient received fluid. Is there anything else you would like to do for this patient?”

Case will end after fluid is initiated, IO is placed, dextrose is given and mannequin is reassessed OR after 10 minutes, whichever comes first

Reassessed – ask for vital signs “is there anything else you would like to do for this patient”

Table 4: Resuscitation checklist

| Category | Action | 1 pt |  | 1 pt |
| --- | --- | --- | --- | --- |
| Basics | - Asks for vital signs - Assesses for malnutrition | Y N  Y N | - Includes HR, BP, RR, Sats | Y N |
| Airway | - Assesses airway/responsiveness | Y N |  |  |
| Breathing | - Assesses breath sounds - Administers oxygen | Y N  Y N | - Specifies amount of O2 to start | Y N |
| Circulation | - Assesses pulse - Assesses capillary refill - Initiates fluids within 5 minutes   **Time to initiation of fluids ______**   - Attempts IO or NG - Rechecks vital signs after fluid initiation - Assesses patient between boluses | Y N  Y N  Y N  Y N  Y N  Y N | - Assesses central pulse - Fluids correct - IO placed correctly - Includes HR, BP, RR, Sats - Auscultates, assesses liver | Y N  Y N  Y N  Y N  Y N |
| Labs | - Checks glucose - Gives dextrose | Y N  Y N | - Gives correct amount of dextrose | Y N |

Abbreviations: Y yes, N no, HR heart rate, BP blood pressure, RR respiratory rate, Sats oxygen saturations, O2 oxygen, IO intraosseous, NG nasogastric

**Data dictionary for checklist**

- Asks for vital signs: 1 pt for asking for any vital signs, 2 pts if includes all four: HR, BP, RR and O2 saturations
- Assesses for responsiveness/airway: Attempts to wake patient with voice and touch OR asks for patient’s neurological status/GCS/sensorium
- Assesses for malnutrition: Asks for MUAC OR asks for patient’s nutrition status OR asks if patient has muscle wasting or hand/foot edema, OR asks for z-score OR acknowledges “patient is very skinny” and chooses slow fluids
- Assesses for breathing: listens to breath sounds OR asks about breathing status
- Administers oxygen: 1 pt if asks for facemask (simple or non-rebreather) or nasal cannula, 2 pts if specifies 1-15 L/min
- Assesses pulse: 1 pt if feels for pulse (any), 2 pts if pulse central (acceptable include femoral, carotid, brachial)
- Assesses capillary refill: Touches fingers or toes OR asks for CRT OR asks for circulation and is given CRT
- Gives fluids: 1 pt if administers bolus of isotonic fluid, initiates MIVF – NO points if boluses hypotonic fluid
- 2 pts if starts 10 – 20 cc/kg D5 1/2NS or D5LR over 1 hour **OR** places NG, give ReSoMal fluid at 5cc/kg every 30 minutes
- Time to initiation of fluids: Time from end of reading case stem to verbalizing fluid initiation – must specify type. Includes initiation of dextrose if correcting hypoglycemia
- Attempts IO – asks for IO and attempts placement. 1 pt if NG placed when IV fails
- Correct placement -- correct site (proximal or distal tibia), remove stylet while stabilizing
- Asks for repeat vital signs: 1 pt if asks for any vital signs, 2 pts if specifies HR, BP SPO2 and RR (give point if patient automatically given all four vital signs)
- Assesses patient following bolus: 1 pt if performs/asks for any element of the physical exam 2 points auscultation, feels for liver
- Checks glucose: asks for glucose (point if asks for electrolytes and given glucose)
- Gives dextrose: 1 pt if asks to correct hypoglycemia or asks to give dextrose, 2 pts if 5cc/kg of D10 (OR 1cc/kg D50, 2cc/kg D25, 10 cc/kg D5

**Debriefing materials**

*Remember: Goal of debriefing is not to lecture, but to facilitate discussion*

**1) Setting the Scene***: “*We are going to spend the next 15-20 minutes debriefing the case with you.”

**2) Reaction**

- “How did that feel?”

**3) Description**

- “Can someone summarize what the case was about from a medical point of view by taking us through what happened? I just want to make sure everyone is on the same page.”
  - “What was the situation when you came in the room?”
  - “What happened next?”

**4) Analysis**

- “What aspects of the case do you think you managed well?”
- “What would you want to do differently next time?”
- Address the key learning points not yet covered using question format outlined in table:
  - ABCs, placing oxygen in shock
  - Identification of shock (what are the different types of shock)
  - Identification of malnutrition
  - Review fluid resuscitation in both malnourished and non-malnourished patients
  - Importance of following vital signs and reassessing patient
  - Alternative access with IO or NG
  - Identifying and treating hypoglycemia

Table 3: Framework for formulating debriefing questions

| **Observation** | **Point of View** | **Question** |
| --- | --- | --- |
| I noticed that… | I liked that… | How do you all see it? |
| I saw that. . . | I was thinking… | What were the team’s priorities at the time? |
| I heard you say… | It seemed to me… | How did the team decide that… |
|  |  | How will you approach that in the future? |
|  |  | Help me understand how the team decided that… |

- *When a team or communication issue has been identified, generalize the discussion*:
  - “Has anyone experienced that before?”
  - “How did you deal with that issue?”
  - “What potential solutions do you see?”
- “Are there any outstanding issues we haven’t discussed yet?

**5) Application/Summarizing**

- Solicit take home messages to achieve and sustain good performance
- Summarize key learning points (each learner first)
